# An instrument for evaluating clinical teaching in Japan: content validity and cultural sensitivity

**DOI:** 10.1186/1472-6920-14-179

**Published:** 2014-08-28

**Authors:** Makoto Kikukawa, Renee E Stalmeijer, Sei Emura, Sue Roff, Albert JJA Scherpbier

**Affiliations:** Department of Medical Education, Kyushu University, 3-1-1 Maidashi Higashi-ku Fukuoka, 81-8582 Kyushu, Japan; Department of Educational Development and Research, Faculty of Health, Medicine and Life Sciences, Maastricht University, Maastricht, The Netherlands; Centre for Graduate Medical Education Development and Research, Saga University Hospital, Saga, Japan; The Centre for Medical Education, Dundee Medical School, Dundee, Scotland; Faculty of Health, Medicine and Life Sciences, Maastricht University, Maastricht, The Netherlands

**Keywords:** Evaluation, Instrument, Clinical teaching, Japan, Culture, Modified Delphi method, Content validity

## Abstract

**Background:**

Many instruments for evaluating clinical teaching have been developed but almost all in Western countries. None of these instruments have been validated for the Asian culture, and a literature search yielded no instruments that were developed specifically for that culture. A key element that influences content validity in developing instruments for evaluating the quality of teaching is culture. The aim of this study was to develop a culture-specific instrument with strong content validity for evaluating clinical teaching in initial medical postgraduate training in Japan.

**Methods:**

Based on data from a literature search and an earlier study we prepared a draft evaluation instrument. To ensure a good cultural fit of the instrument with the Asian context we conducted a modified Delphi procedure among three groups of stakeholders (five education experts, twelve clinical teachers and ten residents) to establish content validity, as this factor is particularly susceptible to cultural factors.

**Results:**

Two rounds of Delphi were conducted. Through the procedure, 52 prospective items were reworded, combined or eliminated, resulting in a 25-item instrument validated for the Japanese setting.

**Conclusions:**

This is the first study describing the development and content validation of an instrument for evaluating clinical teaching specifically tailored to an East Asian setting. The instrument has similarities and differences compared with instruments of Western origin. Our findings suggest that designers of evaluation instruments should consider the probability that the content validity of instruments for evaluating clinical teachers can be influenced by cultural aspects.

**Electronic supplementary material:**

The online version of this article (doi:10.1186/1472-6920-14-179) contains supplementary material, which is available to authorized users.

## Background

Evaluation of undergraduate and postgraduate clinical teaching has received ample attention in the medical education literature, and evaluation instruments have been developed and are being used to monitor teaching in postgraduate programmes [1]. Clinical teaching is essential when residents are trained in clinical practice [2, 3] and is recognised as an important aspect in the postgraduate educational environment [4]. By acting as role models and providing support, clinical teachers can optimize the learning potential of the workplace [5]. There is a considerable body of literature about good clinical teaching ranging from essays to empirical studies [6]. Most instruments for assessing the quality of good clinical teaching have been developed based on the literature and the input of experts and residents/students [7]. Most of these instruments are resident questionnaires [7–9], and different instruments have been developed to fit different educational formats and settings [10–15]. Despite this variety, all currently published instruments originated in Western settings and this begs the question of their transferability to other cultures, considering that “… educational practice is context and culture specific, and research findings in one area may be of limited value to those in different practice settings” [16].

The establishment of the Japanese Council for the Evaluation of Postgraduate Clinical Training, made it necessary to develop an instrument for evaluating clinical teaching. During the development process of the instrument, we decided to take account of the East Asian social background, culture and educational system, all of which have a potential impact on both the definition and evaluation of good clinical teaching [17, 18]. Although it seems logical to develop culture specific evaluation instruments, a literature search revealed no publications describing instruments tailored to the East Asian setting. We therefore decided to adapt an instrument derived from Western questionnaires. Based on our knowledge of Japanese and Western medical education we expected that areas for adaptation would relate to Hofstede’s dimensions of individualism versus collectivism and hierarchical versus egalitarian social relationships. From extensive studies in organizations in different cultural settings, Hofstede derived four dimensions representing cultural values on which organizations are likely to differ, the dimensions of individualism and power distance appeared to be most relevant to the present study [19]. Most Western countries, such as the United States, Great Britain, Canada and the Netherlands rank high on individualism and can also be considered to be a low power distance society, whereas many Asian countries, such as Japan, Hong Kong, Singapore, Thailand, South Korea and Taiwan, value collectivism (low on individualism) and high power distance [19, 20].

Culture has been defined in many ways. One well-known anthropological definition runs as follows:

“*Culture consists in patterned ways of thinking, feeling and reacting, acquired and transmitted mainly by symbols, constituting the distinctive achievements of human groups, including their embodiments in artefacts: the essential core of culture consists of traditional ideas and especially their attached values”* [21]. A key element in the development of instruments for evaluating the quality of teaching which is heavily influenced by cultural factors is content validity, i.e. the congruence between the instrument and what it is designed to measure (good teaching) [22]. Content validity can be determined by surveying experts’ opinions regarding the adequacy and representativeness of items or by including items that are used in similar settings [23]. Considering its sensitivity to cultural factors, we focused on content validity in developing an evaluation instrument tailored to the Japanese culture. After compiling a list of items derived from a literature search and studies of characteristics of good clinical teachers in the Japanese setting [24], we conducted a modified Delphi procedure among different stakeholders to further optimize the content validity of our draft instrument, specifically designed to evaluate clinical teaching during initial residency training in Japan.

## Methods

### Setting

1. Japanese cultural background

Like many East-Asian countries, Japan’s cultural and philosophical background is grounded in Confucianism [25, 26]. In the philosophical and cultural history of East Asia, Confucianism has endured for over a thousand years as the basic social and political value system [27]. In the Confucian philosophy of human nature, propriety of behaviour is the cornerstone of good social relationships, and the study of human nature and human motivations is guided by four principles that directly affect social relationships: humanism, propriety, wisdom and liberal education. Consequently, patterns of interpersonal relationships in East-Asian cultures differ markedly from the individualistic relationship patterns of Western cultures. Basically, Confucian ethics are grounded in relationships and situations rather than in absolute and abstract values. Moreover, cultures influenced by Confucianism are generally characterized by collectivism and a strong power distance and consequently favour communication behaviours that support hierarchical relationships [28]. Confucius contended that the stability of society depends on unequal relationships between people, who have mutual and complementary obligations: the junior partner owes the senior respect and obedience; the senior partner owes the junior partner protection and consideration. In low individualism cultures reactive, Other-directed behaviour is normal while high individualism cultures tend to value extravert and proactive behaviour. The combination of collectivism and hierarchy in East Asian cultures means that individual initiatives, such as those by students, are discouraged and students are far more dependent on teachers than in individualistic, egalitarian cultures where students are encouraged to take initiatives and teachers treat students more or less as equals.

2. Initial postgraduate medical education in Japan

In April 2004, Japan saw the launch of a new two year postgraduate training programme which students can enter after six years of undergraduate medical education and leads to certification of residents’ clinical competence [29]. The programme provides a solid grounding in primary care and general medicine to junior residents regardless of their ultimate choice of specialty. In this sense the programme is comparable to the two-year Foundation programme in the United Kingdom. The development of the programme was triggered by the growing importance attached to evaluation of clinical teaching by the Initial Postgraduate Clinical Training Quality Assurance, established in 2006 by the Japanese Council for the Evaluation of Postgraduate Clinical Training. To ensure continued accreditation as a training hospital, hospitals have to provide evidence of the quality of their clinical teaching [29, 30]. This accountability requirement makes it imperative for training hospitals to evaluate their clinical teaching. With regard to accountability and objective evaluation of postgraduate medical education, Japan is lagging behind Western countries [31–34], which is partly due to the absence of valid evaluation instruments tailored to the Japanese setting [35]. Indeed, most training hospitals in Japan are using evaluation instruments developed by individual residency directors, while the validity and reliability of most of these instruments remain to be established as yet.

### Modified delphi approach

In order to develop an instrument with good content validity for evaluating clinical teaching in the Japanese setting, we conducted a modified Delphi procedure, involving an interactive process designed to establish consensus on specific questions or criteria through systematic collection of informed judgements from professionals in the field [36]. This type of procedure is aimed at achieving consensus among experts in a systematic manner and consists of multiple consultation rounds in which experts indicate their (dis)agreement with statements or concepts [37] *.* Research tells us that the inclusion of different stakeholders in a Delphi procedure promotes acceptance of feedback and effective implementation of the instrument [38]. We therefore included three groups of stakeholders: residents, clinical teachers and educational experts, and although we also considered the inclusion of nurses and clerks, we decided against it, because both in Japan and in other parts of the world, it is not always the case that these groups observe residents and clinical teachers [39]. The modified Delphi procedure has been shown to provide adequate evidence for the content validity of an instrument [40, 41], and we used it because it enables effective consensus building in a situation where published information is inadequate or non-existent [42], and because it has a characteristic that is particularly propitious with regard to Japanese culture, namely that informed judgements are obtained from professionals in a systematic and, more importantly, anonymous manner [36]. This is an important advantage over face-to-face meetings of stakeholders, with the attendant risk of strong personalities dominating the proceedings. Given the hierarchical relationships in Japanese culture, residents are likely to be reluctant to openly disagree with the opinions of their seniors, and consequently in face-to-face sessions with teachers it would be difficult for residents to express their true opinions.

### Preparation for the first delphi round

We started by generating a list of attributes of clinical teachers from a literature search and a previous study [24] in which we explored characteristics of a good clinical teacher as perceived by residents in Japan. In June 2010, the first (M.K.) and third author (E.S.) independently searched PubMed for English-language papers published since 2000 using different combinations of the following keywords: teaching, effectiveness, clinical, assessment, instrument, evaluation, teacher, and inventory. Through a literature search, six articles regarding attributes of effective teachers (one review of the literature article [6], five empirical studies [3, 43–46]), and seven articles of instruments to evaluate clinical teachers (all empirical studies) [4, 11, 12, 14, 15, 47, 48] were identified. All of the articles were reports from Western countries except Zuberi’s Instrument (SETOC) from Pakistan. The two authors (M.K. and E.S.) discussed and agreed on 247 prospective items which were combined with thirty items from our previous study (277 prospective items in total, Additional file 1). We decided that the items of the initial list should relate to observable behaviours as these have been demonstrated to be easier for residents to give feedback on [49]. The items that were considered to have the same meaning were edited from 277 prospective items to an initial list of 52 items and 19 items were excluded as non-observable items through this edition by M.K. and E.S (Additional file 2).

We sent the paper-based list by post to the panellists asking them to rate each item on a four-point scale (1 = unimportant, 2 = of little importance, 3 = important, 4 = very important), suggest changes in wording, detect redundancies and propose additional items. We calculated means and standard deviations and edited the list in accordance with panellists’ comments.

### Recruitment of participants

We selected panellists from the university and the university hospital to ensure representation of three groups of stakeholders: five education experts, twelve clinical teachers and ten residents [50]. During selection, we took into consideration that heterogeneous panels, characterized by members with widely varying personalities and substantially different perspectives on a problem are likely to produce a higher proportion of high quality and highly acceptable solutions than homogeneous groups [51]. The education experts were purposefully selected based on their strong commitment to medical education. They had teaching experience in a variety of medical schools and in the hospital settings. Furthermore they had led professional development activities with regard to teaching and curriculum development. The clinical teachers all had more than seven years’ clinical experience and had worked in a variety of clinical teaching settings (University & Community hospitals). They were purposefully selected from 11 different departs at Saga University hospital (General Medicine, Pediatrics, Emergency, Surgery, Brain Surgery, Urology, Obstetrics and Gynecology, Endocrinology, Dermatology, Neurology, Infection Control). Five First and Five Second Year residents who were training at Saga University Hospitals were randomly selected from the total of 123 residents in the six residency programs of Saga University Hospital (managed by university and community based hospitals).

### Criteria for inclusion of items in the instrument

As there are no standard rules to determine when consensus is reached in a Delphi procedure, we had to decide on criteria to determine at which point consensus was achieved. A number of different approaches was possible: looking at the stability of the response, determining in advance a set number of rounds or setting a percentage at which consensus was achieved [52]. In selecting items for inclusion in the instrument we were guided by the panellists’ ratings and our wish to keep the questionnaire manageable, i.e. not too long, for prospective users. Based on the results of the first round, we selected the 25 items with the highest ratings for resubmission to the panellists in the second round. The results of that round were interpreted using the following criteria [36]:

1. If panellists suggested additional items, an additional Delphi round would be conducted.
2. A standard deviation of <1 was deemed to indicate consensus and considered to be a positive criterion for inclusion in the instrument.

### Ethical approval

This study was approved by the Institutional Review Board of Saga University Hospital. Data was accessible only to the researchers and individual respondents.

## Results

### The first Delphi round

Of 27 panellists, 26 (96%) returned a fully completed questionnaire. Descriptive statistics are presented in Additional file 3. The 25 items with the highest ratings were maintained. In response to suggestions from panellists five items were reworded and eight items that were similar in meaning were combined. Of three new items proposed by panellists, two were included in the list. The third item (“Shows the importance of communication with staff.”) was not included because it was considered to be similar in meaning to item 50 (‘’Makes an effort to establish good relations with medical staff”).

### The second Delphi round

Of 27 panellists, 25 (93%) returned a completed list. The mean ratings and standard deviations are presented in Additional file 1. All items had standard deviations <1.0, so no third round was necessary. As suggested by panellists, item 42 was combined with item 50, and item 26 (‘’Looks up uncertain things together with residents”) was eliminated. As panellists proposed no additional items and made no other negative comments other than the suggestion to eliminate item 26, we concluded that consensus was reached. We had thus obtained a 25-item instrument for evaluating clinical teachers (Additional file 4).

## Discussion

The aim of the present study was to develop, in accordance with previously validated criteria of effective clinical instruction in Japan, a culturally sensitive evaluation instrument tailored to Japanese postgraduate medical education. To achieve this aim, we prepared a draft questionnaire containing items from instruments of Western origin and items resulting from studies of good clinical teaching in Japan. In order to arrive at a usable instrument with good content validity we looked for a method that was sensitive to factors of Japanese culture, strong hierarchy and low individualism in particular. This requirement was met by the modified Delphi method, especially by the anonymity of the procedure allowing all panellists to have their say in the procedure, something which in Japanese culture would be unthinkable in a face-to-face format since it would be unacceptable for junior panellists to express opinions that are opposed to those of their seniors. We think our approach was successful because the resulting instrument appears to reflect the interests and opinions of Japanese residents as elicited in an earlier study. The study was anonymous, although individual panellists were aware of the thoughts of the group, but the modified Delphi procedure prevented any individual from dominating the group.

### Content validity and the impact of cultural factors

The instrument we developed appears to have good content validity based on comparisons with other instruments. For example, ten out of fourteen items (71%) of the Maastricht Clinical Teaching Questionnaire (MCTQ) developed at Maastricht Medical School, the Netherlands [15] are represented in our instrument, and the same holds for ten out of fifteen items (67%) of the Student Evaluation of Teaching in Outpatient Clinics (SETOC) [47], for seventeen out of 28 items (61%) of the Mayo Teaching Evaluation Form (MTEF-28) [1], for twelve out of 32 items (38%) of the Attending Physician Evaluation Form in Department of Medicine, Cook County Hospital [14] and for four of the fifteen items (27%) of The Cleveland Clinic’s Teaching Effectiveness Instrument [12]. In Table 1 10 common items included in most of these instruments are presented.

**Table 1:** **10 common items**

The teacher:	
1.	shows enthusiasm for teaching.
2.	is accessible to residents/students.
3.	provides sufficient support.
4.	treats residents with respect.
5.	actively involves residents in patient care.
6.	sets clear roles for residents.
7.	is a good role model for relationships with medical staff.
8.	is a good role model for doctor-patient relationships.
9.	gives concrete indications of what should be improved.
10.	is a good clinical supervisor at all times.

The items in Table 1 seem to reflect aspects of clinical teaching that are relevant to both Western and Japanese settings and apparently not susceptible to cultural differences.

However, apart from the similarities the instrument we developed bears also witness to culturally determined differences, indicating that the contents of instruments for measuring the quality of clinical teaching should not be uniform for all cultures and countries, but tailored specifically to the culture of the settings in which they are to be used. We will discuss several salient differences between Western instruments and the new Japanese instrument.

Firstly, item 16 in the Japanese instrument: “The teacher demonstrates the importance of safety” is associated with medical risk management, which in Japanese hospitals is currently a major issue, with the Japanese Ministry of Health, Labour and Welfare emphasizing the urgency of addressing this problem. As a result, this topic is included among the objectives of Initial Postgraduate Clinical Training [29], and consequently has found its way into the evaluation questionnaire.

Secondly, the Japanese instrument contains no items relating to independent, active or self-directed learning. The item “promotes self-directed learning” was ranked 38th out of 51 items in the first Delphi round, and consequently eliminated from the instrument. It is quite conceivable that this is an effect of Japanese cultural factors. According to Hofstede [19], in low power distance societies (low hierarchy) teachers tend to treat students as equals and students put value on independence, whereas in high power distance societies, such as Japan, students are dependent on teachers and value conformity. As Japan is a high power distance society due to its Confucian background, stakeholders are only to be expected to give less priority to self-directed learning.

Thirdly, “The teacher shows social common sense” was an item that was added by the panellists. The comparison with other instruments revealed no comparable items and consequently this particular item appears to be quite unique to the Japanese instrument. Teaching social common sense is not a medical subject. It represents a concept that is typical for a high power distance society which, like Japanese society, is steeped in the values of Confucianism, where the junior partner owes the senior respect and obedience. Students treat teachers with respect, even outside the educational setting, and disagreements and confrontations, which might be considered normal in high individualism cultures, are actively avoided [19]. We think that the panellists valued teaching social common sense because, in accordance with the values of their culture, they expect clinical teachers to be respected as seniors while also respecting proper social norms.

During the Delphi procedure, many items were excluded. We believe that those items were not always perceived as unimportant by the panellists (residents, clinical teachers, and educational experts), but the panellists did not emphasize the importance of the items. As a whole, it seems that panelists emphasized the relationships and interaction between residents and clinical teachers, and did not emphasize the content of learning like Evidence Based Medicine. In fact, the previous study showed that Japanese residents seemed to desire interaction with their clinical teachers and they want their teachers to be more accessible. They focused less on the importance of the medical knowledge base of the their teachers [24]. We speculate that this tendency is potentially influenced by collectivism and high power distance because in collectivism society, harmony is emphasized and Confucianism underlines (hierarchical) relationships indicating that residents are less likely to question their teachers’ knowledge base [19]. In addition to that, within Confucianism teachers tend to be considered as Master of a subject, therefor we assume that medical knowledge like EBM was not emphasized in this instrument as much as it might have been. Although the Delphi procedure resulted in a prioritized list of items, we feel that the exclusion of items like “use of guideline or EBM”, “encourages residents to reflect” does not indicate that this topics are not valuable to Japanese learners, they were however not prioritized in the current instrument.

Content validity can be defined as the congruence between the instrument and what it is designed to measure (in this case good clinical teaching in the postgraduate setting). As content validity can be determined by experts’ opinions, we chose to define the concept of “good clinical teaching” in the Japanese clinical postgraduate setting through a consensus procedure among stakeholders. Therefore, we chose a Delphi procedure as the method of achieving consensus of “good teaching” in this study because residents can express their true opinions even under hierarchal relationships. However, further research is still required to investigate what “good teaching” is for the Japanese clinical setting.

### Implications

The main implication of the results of this study is that to enhance the effectiveness of medical education in all cultures, it is of the essence to raise awareness of and sensitivity to cultural differences that impinge on the realm of education research. The instrument we developed is the first to be validated explicitly for the appropriateness of its content for an Asian country. Recognition of the similarities and differences of instruments to be used in Eastern and Western countries will shed light on the importance of consideration and respect for local contexts and cultural backgrounds.

This result may be useful for clinical teachers outside of Asia who are involved in teaching international medical students or postgraduates from an Asian background because they would emphasize these aspects in clinical teaching.

### Limitations

There are several limitations to this study.

1. The number of panellists
  The number of panellists was relatively low. For Delphi studies different numbers of panellists have been reported [53], and while a number of at least 20 panellists has been recommended [54], it is also recommended that the panel should not be too large so as to avoid drop-out. In this study, the response rates of the first and second rounds were 96% (26/27) and 93% (25/27), respectively.
2. Understanding the meaning of items
  It is not inconceivable that panellists may not have quite grasped the meaning of each item of the instrument, as no additional explanations were provided. However, when panellists pointed out that the wording of some items was rather vague, these items were revised for the next round.
3. Translation
  In the translation between Japanese and English, some meanings of the items could not be matched completely. Therefore, it is possible that the nuance of some items has been lost during the translation.
4. A single institution study
  The current study was executed within one educational institution. However, both the experts and the clinical teachers that participated in this study had (teaching) experience in a variety of medical schools and hospital settings. Residents were randomly selected from the six residency programs managed by both university and community based hospitals. Generalizability and transferability of these results to other Asian settings needs to be further investigated.

### Further study

The validity of the Japanese instrument should also be tested in other Asian countries. Similarities and differences between Asian countries may reveal additional effects of cultural aspects. Furthermore, the construct validity should be determined by carrying out both exploratory and confirmatory factor analyses. The generalization (g-coefficient) of the ratings by estimating the number of residents’ ratings required for a reliable rating per individual clinical teacher should also be determined for the Japanese setting as well as for other Asian settings.

## Conclusions

The aim of this study was to develop an instrument with good content validity for evaluating clinical teachers in Japanese postgraduate medical education. This is the first instrument of its kind to be designed and validated for an Asian setting. The instrument has similarities and differences compared with instruments of Western origin, and our findings suggest that designers of evaluation instruments should consider the probability that the content validity of instruments for evaluating clinical teachers can be influenced by cultural aspects.

## Electronic supplementary material

Additional file 1: **277 prospective items.** (DOCX 35 KB)

Additional file 2: **The initial list edited from 277 prospective items.** (DOCX 28 KB)

Additional file 3: **Results for the 52-item draft questionnaire after the first and second Delphi rounds.** Mean ratings (sd) on four-point scale (1 = unimportant; 2 = of little importance; 3:important; 4:very important) and (in bold type) the ranking in order of importance based on the ratings. The items are shown in the order in which they were initially presented to the panel. Information about rewording, combining and elimination of items is provided in the table. (DOCX 26 KB)

Additional file 4: **The final validated instrument.** (DOCX 22 KB)
