# Supplementary material for: An instrument for evaluating clinical teaching in Japan: content validity and cultural sensitivity
Source: BMC Med Educ. 2014 Aug 28;14:179. doi: 10.1186/1472-6920-14-179 (PMC4167259; doi:10.1186/1472-6920-14-179)
Supplement: Supplementary file 1 — Additional file 1: 277 prospective items. (DOCX 35 KB) [file 12909_2014_1010_MOESM1_ESM.docx]

| **Additional file 1.** | **277 prospective items** |
| --- | --- |
| **Authors** | **Prospective items** |
| Sutkin[6] | Demonstrates medical/clinical knowledge |
|  | Demonstrates clinical technical skills/competence, clinical reasoning |
|  | Show enthusiasm for medicine |
|  | Models a close doctor-patient relationship |
|  | Exhibits professionalism |
|  | Is scholarly |
|  | Values teamwork and has collegial skills |
|  | Is experienced |
|  | Demonstrates skills in leadership and/or administration |
|  | Accepts uncertainty in medicine |
|  | Maintains positive relationships with students and a supportive learning environment |
|  | Demonstrates enthusiasm for teaching |
|  | Is accessible/available to students |
|  | Provides effective explanations, answers to questions, and demonstrations |
|  | Provides feedback and formative assessment |
|  | Is organized and communicates objectives |
|  | Demonstrates knowledge of teaching skills, methods, principles, and their application |
|  | Stimulate students' interest in learning and/or subject |
|  | Stimulate or inspires trainees' thinking |
|  | Encourages trainees' active involvement in clinical work |
|  | Provide indivisual attention to students |
|  | Demonstrate commitment to improving of teaching |
|  | Actively involves students |
|  | Demonstrates learner assessment/evaluation skills |
|  | Uses questioning skills |
|  | Stimulates trainees' reflective practice and assessment |
|  | Teacher professionalism |
|  | Is dynamic, enthusiastic, and engaging |
|  | Emphasizes observation |
|  | Communication skills |
|  | Acts as role model- other |
|  | Is an enthusiastic person in general |
|  | Is personable |
|  | Is compassionate/empathetic |
|  | Respects others |
|  | Displays honesty/integrity |
|  | Has wisdom, intelligence, common sense, and good judgment |
|  | Appreciate culture and different cultural backgrounds |
|  | Consider others' perspectives, viewpoints |
|  | Is patient |
|  | Balance professional and personal life |
|  | Is perceived as a virtuous person and a globally good person |
|  | Maintains health, appearance, and hygiene |
|  | Is modest and humble |
|  | Has a good sense of humor |
|  | Is responsible and conscientious |
|  | Is imaginative |
|  | Has self-insight, self-knowledge, and is reflective |
|  | Is altruistic |
| Ker[43] | has a high level of operative and clinical competence |
|  | shows interest in the trainee |
|  | treats all junior staff and nursing staff with respect |
|  | knows own limitations |
|  | is someone the trainee can respect clinically and professionally |
|  | knows when to let the trainee do the operation on his/her own |
|  | is approachable |
|  | engages self confidence in the trainee |
|  | demonstrates logical assessment of emergency admissions |
|  | demonstrates the importance of safety |
| Martens[44] | Discuss students’ preparatory reading in a constructive, non-judgmental manner |
|  | Be aware of students’ level of knowledge and understanding |
|  | Demonstrate skills step-by-step |
|  | Ask probing questions |
|  | Embed skills training in underlying basic science knowledge |
|  | Help students understand and correct their mistakes |
|  | Stimulate collaboration |
|  | Stimulate contextual learning |
|  | Strike a good balance between questioning and lecturing |
|  | Give constructive positive feedback and explain negative feedback |
|  | Show links between physical examination and clinical practice |
|  | Explain the implications of possible outcomes of physical examination |
|  | Ask for critical feedback on teaching and training sessions |
|  | Treat students as equals |
|  | Respect students’ personal integrity |
|  | Use male rather than female models |
|  | Invite students to volunteer rather than select them |
|  | Show enthusiasm |
|  | Two-way integration of skills training with concurrent curricular components |
|  | Structured training sessions |
|  | Delivery of a summary at the end of a training session |
|  | Sufficient knowledge of the subject on the part of the teacher |
|  | Proper preparation for the training session on the part of the teacher |
|  | Good time management of a session |
| Huggett[45] | Demonstrates professional expertise |
|  | Actively engages students in learning |
|  | Creates a positive environment for teaching and learning |
|  | Demonstrates collegiality and professionalism |
|  | Discusses career-related topics and concerns |
| Kisiel[3] | Kindness and sensitivity |
|  | Teacher-learner relationships |
|  | Personality and style |
|  | Discussing versus delivering content |
|  | Modeling self-directed learning |
|  | Autonomy |
|  | Feedback |
|  | Diagnosing the learner |
|  | Expertise |
|  | Preferential staffing |
|  | Irreverent teaching |
|  | General |
| Yeates[46] | Is clinically up-to-date and competent |
|  | Identifies patients who are appropriate for student teaching |
|  | Able to deliver opportunistic teaching from available clinical material |
|  | Can appropriately modify own teaching in response to evaluation |
|  | Acquires consent from patients who have been identified as appropriate for teaching |
|  | Able to clearly communicate goals and outcomes |
|  | Can teach in accordance with goals and outcomes |
|  | Keeps (approximately) to time during planned teaching |
|  | Within time constraints, is receptive to questions and discussion |
|  | Possesses good communication and listening skills |
|  | Shows an appropriate amount of enthusiasm while teaching (considering subject and teaching method) |
|  | When appropriate, allows students to be involved with (rather than passively observe) clinical learning opportunities |
|  | Gives positive and negative feedback to students on their performance of a task |
|  | Delivers a volume of content appropriate to the length of the session |
|  | Displays compassion and empathy towards patients |
|  | Avoids sexism or racism |
|  | Avoids deliberate belittling, insensitivity, humiliation and inappropriate interruption |
|  | Attends punctually or arranges cover if absence required, or informs students if not possible to arrange cover |
|  | Manifests an even temper and is patient and polite |
|  | Avoids inappropriate disclosure of information relating to students |
|  | Avoids displays of arrogance or pomposity |
|  | Displays tolerance towards cultural issues and student beliefs without compromising institutional values |
|  | Avoids favoritism |
|  | Is an appropriate role model of clinical practice when teaching in a clinical environment |
|  | Avoids inappropriate use of sense of humor |
|  | Knows who to contact if concerned about a student |
|  | Promotes the necessity of gaining consent from patients to involve them in teaching, and maintaining their confidentiality |
| Beckman[11] | Made learners feel comfortable asking questions on rounds |
|  | Allowed learners to present without frequent interruption |
|  | Never ordered tests without telling the learner |
|  | Expressed respect for learners |
|  | Treated his or her residents kindly |
|  | Was a good role model of a caring doctor |
|  | Showed enthusiasm for his/her work/learners |
|  | Started and finished rounds on time |
|  | Did not delay rounds to write lengthy notes |
|  | Discouraged external interruptions |
|  | Stated goals and expectations of the team |
|  | Stated relevance of goals to learners |
|  | Prioritized goals |
|  | Repeated goals periodically |
|  | Provided didactic teaching on non-admission days |
|  | Gave justification before changing learner’s plan |
|  | Used blackboard or other visual aids |
|  | Asked learner to discuss differential diagnosis on most patients |
|  | Asked learners to discuss alternative management options on most patients |
|  | Asked learners to demonstrate physical exam skills on rounds |
|  | Evaluated learner’s knowledge of factual medical information |
|  | Gave learners regular, useful feedback on their performance |
|  | Gave negative (corrective) feedback to learners |
|  | Explained to learners why they were correct or incorrect |
|  | Offered learners suggestions for improvement |
|  | Encouraged learners to pursue the literature to answer specific questions |
|  | Motivated learners to learn on their own |
|  | Encouraged learners to do outside reading |
| Copeland[12] | Establishes a good learning environment (approachable, nonthreatening, enthusiastic, etc.) |
|  | Stimulates me to learn independently |
|  | Allows me autonomy appropriate to my level/experience/competence |
|  | Organizes time to allow for both teaching and care giving |
|  | Offers regular feedback (both positive and negative) |
|  | Clearly specifies what I am expected to know and do during this training period |
|  | Adjusts teaching to my needs (experience, competence, interest, etc.) |
|  | Asks questions that promote learning (clarifications, probes, Socratic questions, reflective questions, etc.) |
|  | Gives clear explanations/reasons for opinions, advice, actions, etc. |
|  | Adjusts teaching to diverse settings (bedside, view box, OR, exam room, microscope, etc.) |
|  | Coaches me on my clinical/technical skills (interview, diagnostic, examination, procedural, lab, etc.) |
|  | Incorporates research data and/or practice guidelines into teaching |
|  | Teaches diagnostic skills (clinical reasoning, selection/interpretation of tests, etc.) |
|  | Teaches effective patient and/or family communication skills |
|  | Teaches principles of cost-appropriate care (resource utilization, etc.) |
| Sue [4] Extracted items regarding teaching | My clinical teachers set clear expectations. |
|  | I have protected educational time in this post. |
|  | I have good clinical supervision at all times. |
|  | My clinical teachers have good communication skills. |
|  | My clinical teachers are enthusiastic. |
|  | I get regular feedback from seniors. |
|  | My clinical teachers are well organized. |
|  | I have enough clinical learning opportunities for my needs. |
|  | My clinical teachers have good teaching skills. |
|  | My clinical teachers are accessible. |
|  | My clinical teachers encourage me to be an independent learner. |
|  | The clinical teachers provide me with good feedback on my strengths and weaknesses. |
|  | My clinical teachers promote an atmosphere of mutual respect |
| Smith[47] | demonstrated a board knowledge of medicine. |
|  | was up to date. |
|  | saw all patients every day. |
|  | independently evaluated each patient. |
|  | reviewed the care plan of each patient with the team every day. |
|  | contributed additional clinical information or advice when needed. |
|  | expected me to develop a thorough differential diagnosis and management plan for each active problem. |
|  | helped speak with consultations and helped arrange tests in order to provide the best care for the patients. |
|  | conducted post-call rounds in an effective and efficient manner. |
|  | required me to be an active decision-maker in patient care, rather than always following the attending's lead. |
|  | made sure the teaching sessions pertained to patient problems. |
|  | effectively taught interviewing and communication skills. |
|  | effectively taught physical examination skills. |
|  | was always explicit about his or her reasoning when discussing clinical decisions. |
|  | expected me to be an active learner, by requiring me to ask focused questions, finding the best literature, and sharing my findings with the team. |
|  | personally modeled active, continuous learning by asking questions, searching the literature, and sharing his or her findings. |
|  | expected me to commit to a working diagnosis. |
|  | personally modeled committing to a working diagnosis. |
|  | expected me to incorporate the best evidence from the literature with the patient's unique circumstances and preferences. |
|  | personally modeled incorporating the best evidence from the literature with the patient's unique circumstances and preferences. |
|  | provided ongoing feedback on my history-taking skills, physical exam skills, written documentation, oral presentations, and clinical reasoning. |
|  | clearly stated his or her expectations for my performance this rotation. |
|  | provided specific, detailed mid-rotation feedback. |
|  | treated the patients with respect. |
|  | treated me with respect. |
|  | released me for all scheduled conferences. |
|  | encouraged me to call him or her at any time for any reason. |
|  | was sensitive to the emotional, economic, social, and cultural aspects of the patients' illnesses. |
|  | would like to work with this attending again? |
|  |  |
|  | We did not include the items below because they do not always fit to Japanese setting. |
|  | Aside from post-call rounds, did the attending participate in bedside work rounds (7:30-9 am) for the first week on service and at least twice a week thereafter? |
|  | Did work rounds always take place at the bedside? |
|  | What's your best estimate for the number of teaching sessions with the team and the attending? (Consider a "teaching session" to mean a minimum of 45 minutes devoted to education.) |
| Zuberi[48] | Rapport with members of the health care team |
|  | Demonstration of signs and symptoms |
|  | Pace of teaching on the patients in the clinic (Balancing time for patient care and teaching) |
|  | Setting of expectations regarding the clinics (responsibilities, assignments, grading etc.) |
|  | Observation of your data gathering skills |
|  | Practical application of Instructor’s knowledge to patient problems |
|  | Discussions on your assessment and management plan |
|  | Identification of your strengths |
|  | Clear emphasis on key points of the case |
|  | Use of ‘‘evidence’’ to make decisions regarding diagnosis, tests or therapy |
|  | Identification of areas needing improvement |
|  | Probing your understanding by asking questions of ‘‘Why’’ or ‘‘What if’’ or ‘‘Suppose’’ or ‘‘What else’’ variety. |
|  | Observation of your patient education skills |
|  | Ability to stimulate your interest in new issues |
|  | Rate the idea of doing more clinics with the instructor |
| Sue [49]  Extracted from "Students' perception of teaching" | The teacher is sufficiently concerned to develop my confidence |
|  | The teaching encourages me to be an active learner |
|  | The teacher is well focused |
|  | The teaching is sufficiently concerned to develop my competence |
|  | I am clear about the learning objectives of the course |
|  | The teaching is often stimulating |
| Renee[15] | Consistently demonstrated how to perform clinical skills. |
|  | Created sufficient opportunities for me to observe him/her. |
|  | Served as a role model as to the kind of doctor I would like to become. |
|  | Gave useful feedback during or immediately after direct observation of my patient encounters. |
|  | Adjusted his/her teaching activities to my level of experience. |
|  | Offered me sufficient opportunities to perform activities independently. |
|  | Asked me to provide a rationale for my actions. |
|  | Asked me questions aimed at increasing my understanding. |
|  | Stimulated me to explore my strengths and weaknesses. |
|  | Encouraged me to formulate learning goals. |
|  | Encouraged me to pursue my learning goals. |
|  | Created a safe learning environment. |
|  | Was genuinely interested in me as a student. |
|  | Showed that he/she respected me. |
| Makoto [24] | Provided sufficient support |
|  | Presented residents with chances to think |
|  | Provided feedback |
|  | Provided specific indications of areas needing improvement |
|  | Was accessible |
|  | Provided residents with opportunities to practice |
|  | Established clear roles for residents |
|  | Did not criticize residents’ personalities |
|  | Treated individual residents equally |
|  | Provided opportunities for consultation |
|  | Did not get angry with residents |
|  | Was an enthusiastic teacher |
|  | Acknowledged residents as doctors |
|  | Actively involved residents in patient care |
|  | Provided clear explanations |
|  | Treated residents as equals |
|  | Thought about areas of uncertainty together with residents |
|  | Looked up information together with residents |
|  | Did not compare the ability of a resident with that of other residents |
|  | Was not boring |
|  | Established clear goals |
|  | Showed the next step |
|  | Provided references for further learning |
|  | Assessed residents |
|  | Was enthusiastic about patient care |
|  | Did not get angry with patients |
|  | Demonstrated reasoning processes |
|  | Did not display bad manners |
|  | Established good relationships with medical staff |
|  | Did not pretend to know everything |
