# Supplementary material for: An instrument for evaluating clinical teaching in Japan: content validity and cultural sensitivity
Source: BMC Med Educ. 2014 Aug 28;14:179. doi: 10.1186/1472-6920-14-179 (PMC4167259; doi:10.1186/1472-6920-14-179)
Supplement: Supplementary file 2 — Additional file 2: The initial list edited from 277 prospective items. (DOCX 28 KB) [file 12909_2014_1010_MOESM2_ESM.docx]

| **Additional file 2.** | **The initial list edited from 277 prospective items** |
| --- | --- |
| **Initial list** | **Edition from prospective items** |
| 1. Demonstrates awareness of and sensitivity to residents’ learning needs. | Adjusts teaching to my needs (experience, competence, interest, etc.), Shows interest in the trainee, kindness and sensitivity, I have enough clinical learning opportunities for my needs |
| 2. Stimulates residents' interest in learning and/or subject. | Stimulate students' interest in learning and/or subject, Invite students to volunteer rather than select them, Ability to stimulate your interest in new issues, the teaching is often stimulating |
| 3. Shows enthusiasm for teaching. | My clinical teachers are enthusiastic, Show enthusiasm, demonstrates enthusiasm for teaching, Shows an appropriate amount of enthusiasm while teaching (considering subject and teaching method), Showed enthusiasm for his/her work/learners, Establishes a good learning environment (approachable, nonthreatening, enthusiastic, etc.), Was an enthusiastic teacher, Is an enthusiastic person in general |
| 4. Promotes self-directed learning. | The teaching encourages me to be an active learner, Engages self confidence in the trainee, modeling self-directed learning, Discouraged external interruptions, Motivated learners to learn on their own, Stimulates me to learn independently, Expected me to be an active learner, by requiring me to ask focused questions, finding the best literature, and sharing my findings with the team, Personally modeled active, continuous learning by asking questions, searching the literature, and sharing his or her findings, The teacher is sufficiently concerned to develop my confidence, Encouraged me to pursue my learning goals, My clinical teachers encourage me to be an independent learner |
| 5. Actively involves residents in patient care. | Actively involved residents in patient care, Actively involves students, Stimulate collaboration, Actively engages students in learning |
| 6. Stimulates residents to think critically when solving a problem. | Stimulate or inspires trainees' thinking, Ask probing questions, Probing your understanding by asking questions of ‘‘Why’’ or ‘‘What if’’ or ‘‘Suppose’’ or ‘‘What else’’ variety, Asked me questions aimed at increasing my understanding |
| 7. Requires residents to be active decision-makers in patient care, rather than to always follow the attending's lead. | Required me to be an active decision-maker in patient care, rather than always following the attending's lead, Autonomy, When appropriate, allows students to be involved with (rather than passively observe) clinical learning opportunities, Asked learners to discuss alternative management options on most patients, Expected me to develop a thorough differential diagnosis and management plan for each active problem, Expected me to commit to a working diagnosis, My clinical teachers encourage me to be an independent learner |
| 8. Expects residents to incorporate in clinical reasoning the best evidence from the literature as well as the patient's unique circumstances and preferences. | Encouraged learners to pursue the literature to answer specific questions, Expected me to incorporate the best evidence from the literature with the patient's unique circumstances and preferences, Use of ‘‘evidence’’ to make decisions regarding diagnosis, tests or therapy, Asked me to provide a rationale for my actions, Stimulate contextual learning, |
| 9. Gives concrete indications as to what should be improved. | Offered learners suggestions for improvement, Help students understand and correct their mistakes, Give constructive positive feedback and explain negative feedback, Feedback, Gives positive and negative feedback to students on their performance of a task, Gave learners regular, useful feedback on their performance, Gave negative (corrective) feedback to learners, Offers regular feedback (both positive and negative), Provided ongoing feedback on my history-taking skills, physical exam skills, written documentation, oral presentations, and clinical reasoning, Identification of areas needing improvement, The teaching is sufficiently concerned to develop my competence, Gave useful feedback during or immediately after direct observation of my patient encounters, Provided feedback, Provided specific indications of areas needing improvement, I get regular feedback from seniors, The clinical teachers provide me with good feedback on my strengths and weaknesses |
| 10. Does not provide negative feedback in front of others. | Respects others |
| 11. Does not criticize residents’ personalities. | Did not criticize residents’ personalities, Avoids sexism or racism, Avoids deliberate belittling, Insensitivity, Humiliation and inappropriate interruption |
| 12. Treats residents with respect. | Treated me with respect, Consider others' perspectives, viewpoints, Treats all junior staff and nursing staff with respect, Discuss students’ preparatory reading in a constructive, non-judgmental manner, Treat students as equals, Respect students’ personal integrity, Teacher-learner relationships, Avoids deliberate belittling, insensitivity, humiliation and inappropriate interruption, Manifests an even temper and is patient and polite, Avoids inappropriate disclosure of information relating to students, Avoids favoritism, Avoids inappropriate use of sense of humor, Expressed respect for learners, Showed that he/she respected me, Acknowledged residents as doctors, Did not compare the ability of a resident with that of other residents, My clinical teachers promote an atmosphere of mutual respect, Is empathetic |
| 13. Does not teach residents in an angry voice. | Did not get angry with residents, Treated his or her residents kindly, Establishes a good learning environment (approachable, nonthreatening, enthusiastic, etc.) |
| 14. Displays his/her reasoning processes. | Demonstrated reasoning processes, Demonstrates clinical technical skills/competence, clinical reasoning, Show links between physical examination and clinical practice, Explain the implications of possible outcomes of physical examination, Was always explicit about his or her reasoning when discussing clinical decisions |
| 15. Encourages residents to call him or her at any time for any reason. | Encouraged me to call him or her at any time for any reason, provided opportunities for consultation, Teacher professionalism |
| 16. Contributes additional clinical information, gives advice about diagnosis and management plan for each active problem. | Contributed additional clinical information or advice when needed, Discussing versus delivering content, Asked learner to discuss differential diagnosis on most patients, Teaches diagnostic skills (clinical reasoning, selection/interpretation of tests, etc.), Teaches principles of cost-appropriate care (resource utilization, etc.), Showed the next step |
| 17. Takes sufficient time to discuss matters with residents. | Discussions on your assessment and management plan, Is patient, Keeps (approximately) to time during planned teaching, reviewed the care plan of each patient with the team, Conducted post-call rounds in an effective and efficient manner, Made sure the teaching sessions pertained to patient problems |
| 18. Is accessible to residents. | Is accessible/available to students, Is approachable within time constraints, Is receptive to questions and discussion, Made learners feel comfortable asking questions on rounds, Establishes a good learning environment (approachable, nonthreatening, enthusiastic, etc.), Was accessible, My clinical teachers are accessible, Possesses good communication and listening skills |
| 19. Teaches residents in accordance with their level of training. | Adjusted his/her teaching activities to my level of experience, knows when to let the trainee do the operation on his/her own, be aware of students’ level of knowledge and understanding, demonstrate skills step-by-step, identifies patients who are appropriate for student teaching, can teach in accordance with goals and outcomes, allows me autonomy appropriate to my level/experience/competence, coaches me on my clinical/technical skills (interview, diagnostic, examination, procedural, lab, etc.) |
| 20. Provides sufficient support. | Provided sufficient support, Maintains positive relationships with students and a supportive learning environment, Two-way integration of skills training with concurrent curricular components, Creates a positive environment for teaching and learning, Attends punctually or arranges cover if absence required, or informs students if not possible to arrange cover, Did not delay rounds to write lengthy notes, Consistently demonstrated how to perform clinical skills, Created a safe learning environment |
| 21. Gives individual attention to residents. | Provide individual attention to students, Observation of your data gathering skills, Identification of your strengths, Was genuinely interested in me as a student, Treated individual residents equally |
| 22. Encourages residents to reflect. | Stimulates trainees' reflective practice and assessment, Asks questions that promote learning (clarifications, probes, Socratic questions, reflective questions, etc.), Stimulated me to explore my strengths and weaknesses |
| 23. Is a good clinical supervisor at all times. | I have good clinical supervision at all times, Displays tolerance towards cultural issues and student beliefs without compromising institutional values, Coaches me on my clinical /technical skills (interview, diagnostic, examination, procedural, lab, etc.), Observation of your patient education skills, Created sufficient opportunities for me to observe him/her |
| 24. Demonstrates the importance of safety. | Demonstrates the importance of safety |
| 25. Answers questions clearly and precisely. | Gives clear explanations/reasons for opinions, advice, actions, etc |
| 26. Teaches speculatively about areas of uncertainty and together with residents looks up information about areas of uncertainty. | Thought about areas of uncertainty together with residents, Looked up information together with residents |
| 27. Helps residents to speak during consultations and helps arrange tests in order to provide the best care for patients. | Helped speak with consultations and helped arrange tests in order to provide the best care for the patients, Discussing versus delivering content, Gave justification before changing learner’s plan |
| 28. Releases residents for all scheduled conferences. | Released me for all scheduled conferences, I have protected educational time in this post. |
| 29. Incorporates research data and/or practice guidelines into teaching. | Incorporates research data and/or practice guidelines into teaching, Embed skills training in underlying basic science knowledge |
| 30. Provides further reference for follow-up learning. | Provided references for further learning, Encouraged learners to do outside reading, personally modeled active, Continuous learning by asking questions, searching the literature, and sharing his or her findings |
| 31. Explains clearly. | Provided clear explanations, Irreverent teaching, Gives clear explanations/reasons for opinions, advice, actions, etc, Clear emphasis on key points of the case, The teacher is well focused, Preferential staffing |
| 32. Explains clearly during performance of a task which aspects are important and why. | Explained to learners why they were correct or incorrect, Provides effective explanations, answers to questions, and demonstrations, Irreverent teaching, Provided didactic teaching on non-admission days, Provided specific, detailed mid-rotation feedback |
| 33. Gives residents opportunities to think. | Presented residents with chances to think |
| 34. Gives residents opportunities to practice. | Provided residents with opportunities to practice, Asked learners to demonstrate physical exam skills on rounds, Effectively taught physical examination skills, Offered me sufficient opportunities to perform activities independently |
| 35. Has good teaching skills. | My clinical teachers have good teaching skills, Demonstrates knowledge of teaching skills, methods, principles, and their application, Uses questioning skills, Strike a good balance between questioning and lecturing, Structured training sessions, Delivery of a summary at the end of a training session, Good time management of a session, Able to deliver opportunistic teaching from available clinical material, Delivers a volume of content appropriate to the length of the session, Started and finished rounds on time, Organizes time to allow for both teaching and care giving, Asks questions that promote learning (clarifications, probes, Socratic questions, reflective questions, etc.), Adjusts teaching to diverse settings (bedside, view box, OR, exam room, microscope, etc.), Pace of teaching on the patients in the clinic (Balancing time for patient care and teaching), Was not boring, My clinical teachers have good communication skills, My clinical teachers are well organized, Possesses good communication and listening skills, Emphasizes observation |
| 36. Demonstrates commitment to improving teaching. | Demonstrate commitment to improving of teaching, Ask for critical feedback on teaching and training sessions, Can appropriately modify own teaching in response to evaluation, Is responsible and conscientious, Has self-insight, self-knowledge, and is reflective, |
| 37. Sets clear roles for residents. | Established clear roles for residents, Setting of expectations regarding the clinics (responsibilities, assignments, grading etc.), My clinical teachers set clear expectations |
| 38. Discusses goals with residents. | Established clear goals, Stated goals and expectations of the team, Stated relevance of goals to learners, Encouraged me to formulate learning goals |
| 39. Clearly specifies what the resident is expected to know and do during the training period. | Clearly specifies what I am expected to know and do during this training period, Clearly stated his or her expectations for my performance this rotation, Is organized and communicates objectives, Discussing versus delivering content, Prioritized goals, Repeated goals periodically, I am clear about the learning objectives of the course |
| 40. Demonstrates skills for learner assessment/evaluation. | Demonstrates learner assessment/evaluation skills, Provides feedback and formative assessment, Diagnosing the learner, Evaluated learner’s knowledge of factual medical information, Gave negative (corrective) feedback to learners, Offers regular feedback (both positive and negative), Assessed residents, I get regular feedback from seniors. |
| 41. Shares up-to-date knowledge of developments in the field. | Was up to date, Personally modeled active, continuous learning by asking questions, searching the literature, and sharing his or her findings |
| 42. Encourages residents to discuss the care plan of each patient within the team. | Encourages trainees' active involvement in clinical work, Allowed learners to present without frequent interruption, Never ordered tests without telling the learner |
| 43. Is a scholar. | Is scholarly |
| 44. Is a good role model of doctor-patient relationships. | Models a close doctor-patient relationship, Acts as role model- other, is someone the trainee can respect clinically and professionally, Displays compassion and empathy towards patients, Served as a role model as to the kind of doctor I would like to become, Was enthusiastic about patient care |
| 45. Teaches effective communication skills. | Effectively taught interviewing and communication skills, Teaches effective patient and/or family communication skills, Communication skills |
| 46. Treats patients with respect. | Treated the patients with respect, Displays compassion and empathy towards patients, Avoids displays of arrogance or pomposity, Was a good role model of a caring doctor, Saw all patients every day, Independently evaluated each patient, Was sensitive to the emotional, economic, social, and cultural aspects of the patients' illnesses, Did not get angry with patients, Exhibits professionalism |
| 47. Is competent. | Demonstrates clinical technical skills/competence, clinical reasoning, Demonstrates medical/clinical knowledge, Is experienced, Has a high level of operative and clinical competence, Is someone the trainee can respect clinically and professionally, Demonstrates logical assessment of emergency admissions, Sufficient knowledge of the subject on the part of the teacher, Demonstrates professional expertise, Expertise, Is clinically up-to-date and competent, Is an appropriate role model of clinical practice when teaching in a clinical environment, Demonstrated a board knowledge of medicine, Personally modeled committing to a working diagnosis, Personally modeled incorporating the best evidence from the literature with the patient's unique circumstances and preferences, Demonstration of signs and symptoms, Practical application of Instructor’s knowledge to patient problems |
| 48. Shows enthusiasm for medicine. | Show enthusiasm for medicine |
| 49. Does not pretend to know all things. | Did not pretend to know everything, Displays honesty/integrity/knows own limitations, Accepts uncertainty in medicine |
| 50. Makes an effort to establish good relations with medical staff. | Established good relationships with medical staff, Values teamwork and has collegial skills, Demonstrates skills in leadership and/or administration, Treats all junior staff and nursing staff with respect, Rapport with members of the health care team, Exhibits professionalism |
| 51. Maintains health, appearance and hygiene. | Maintains health, appearance, and hygiene, Did not display bad manners, Is personable, |
| 52. I would like to work with this attending physician again. | Would like to work with this attending again?, Rate the idea of doing more clinics with the instructor |
|  |  |
| Excluded non-observable items | Use male rather than female models, Is imaginative, Personality and style, Is dynamic, enthusiastic, and engaging, Has wisdom, intelligence, common sense, and good judgment, Appreciate culture and different cultural backgrounds, Balance professional and personal life, Is perceived as a virtuous person and a globally good person, Is modest and humble, Has a good sense of humor, Is altruistic, Proper preparation for the training session on the part of the teacher, Demonstrates collegiality and professionalism, Discusses career-related topics and concerns, Used blackboard or other visual aids, General, Acquires consent from patients who have been identified as appropriate for teaching, Knows who to contact if concerned about a student, Promotes the necessity of gaining consent from patients to involve them in teaching, and maintaining their confidentiality |
