# Supplementary material for: An instrument for evaluating clinical teaching in Japan: content validity and cultural sensitivity
Source: BMC Med Educ. 2014 Aug 28;14:179. doi: 10.1186/1472-6920-14-179 (PMC4167259; doi:10.1186/1472-6920-14-179)
Supplement: Supplementary file 3 — Additional file 3: Results for the 52-item draft questionnaire after the first and second Delphi rounds. Mean ratings (sd) on four-point scale (1 = unimportant; 2 = of little importance; 3:important; 4:very important) and (in bold type) the ranking in order of importance based on the ratings. The items are shown in the order in which they were initially presented to the panel. Information about rewording, combining and elimination of items is provided in the table. (DOCX 26 KB) [file 12909_2014_1010_MOESM3_ESM.docx]

| **Table 3.** Results for the 52-item draft questionnaire after the first and second Delphi rounds. Mean ratings (sd) on four-point scale (1=unimportant; 2=of little importance; 3:important; 4:very important) and (in bold type) the ranking in order of importance based on the ratings. The items are shown in the order in which they were initially presented to the panel. Information about rewording, combining and elimination of items is provided in the table. | | |
| --- | --- | --- |
| **The teacher:** | **RESULTS**  Mean rating (sd)  **Ranking after Delphi round** | |
|  | **First Delphi round** | **Second Delphi round** |
| 1. Demonstrates awareness of and sensitivity to residents’ learning needs. | 2.68 (0.802)  **39** | eliminated |
| 2. Stimulates residents' interest in learning and/or subject. | 1. (0.764)   **22** | - 1. (0.900)   **25** |
| 3. Shows enthusiasm for teaching. | 3.56 (0.507)  **3** | 3.44 (0.580)  **5** |
| 4. Promotes self-directed learning. | 2.72 (0.792)  **38** | eliminated |
| 5. Actively involves residents in patient care. | 3.16 (0.943)  **18** | 3.16 (0.750)  **15** |
| 6. Stimulates residents to think critically when solving a problem. | 2.96 (0.790)  **24** | 2.88 (0.600)  **22** |
| 7. Requires residents to be active decision-makers in patient care, rather than to always follow the attending's lead. | 2.84 (0.688)  **32** | eliminated |
| 8. Expects residents to incorporate in clinical reasoning the best evidence from the literature as well as the patient's unique circumstances and preferences. | 2.52 (0.963)  **50** | eliminated |
| 9. Gives concrete indications as to what should be improved. | 3.48 (0.586)  **5** | 3.40 (0.710)  **6** |
| 10. Does not provide negative feedback in front of others. | 3.24 (0.779)  **15** | Items 10-13 were combined into a new item 10 in the second round  3.60 (0.710)  **2** |
| 11. Does not criticize residents’ personalities. | 3.60 (0.707)  **2** |  |
| 12. Treats residents with respect. | 3.20 (0866)  **16** |  |
| 13. Does not teach residents in an angry voice. | 3.48 (0.823)  **6** |  |
| Combination of 10-13 in second round because of similarity of meaning.  10. Treats resident with respect (does not criticize, does not teach residents in an angry voice, does not provide negative feedback in front of others). |  |  |
| 14. Displays his/her reasoning processes. | 3.44 (0.651)  **7** | 3.48 (0.710)  **4** |
| 15. Encourages residents to call him or her at any time for any reason. | 3.16 (0.898)  19 | 2.92 (0.760)  21 |
| 16. Contributes additional clinical information, gives advice about diagnosis and management plan for each active problem. | 3.36 (0.757)  **10** | 3.08 (0.700)  **17** |
| 17. Takes sufficient time to discuss matters with residents. | 2.88 (0.833)  **29** | eliminated |
| 18. Is accessible to residents. | 3.56 (0.507)  **4** | 3.36 (0.760)  **7** |
| 19. Teaches residents in accordance with their level of training. | 2.80 (0.866)  **34** | eliminated |
| 20. Provides sufficient support. | 2.96 (0.790)  **24** | 3.00 (0.660)  **19** |
| 21. Gives individual attention to residents. | 2.88 (0.666)  **30** | eliminated |
| 22. Encourages residents to reflect. | 2.67 (0.702)  **43** | eliminated |
| 23. Is a good clinical supervisor at all times. | 3.28 (0.891)  **12** | 3.60 (0.580)  **1** |
| 24. Demonstrates the importance of safety. | 3.28 (0.891)  **13** | 3.32 (0.630)  **10** |
| 25. Answers questions clearly and precisely. | 2.62 (0.824)  **47** | eliminated |
| 26. Teaches speculatively about areas of uncertainty and together with residents looks up information about areas of uncertainty.  The item was reworded in response to panellists’ suggestions into:  26: Thinks speculatively about areas of uncertainty with residents.  and | 2.67 (0.917)  **44** | 3.16 (0.700)  **14** |
| 26. Looks up uncertain things together with residents. |  | 2.29 (0.860)  **27**  Eliminated based on opinions of several panellists |
| 27. Helps residents to speak during consultations and helps arrange tests in order to provide the best care for patients. | 2.52 (0.918)  **51** | eliminated |
| 28. Releases residents for all scheduled conferences. | 2.68 (0.945)  **40** | eliminated |
| 29. Incorporates research data and/or practice guidelines into teaching. | 2.80 (0.764)  **35** | eliminated |
| 30. Provides further reference for follow-up learning. | 2.64 (0.810)  **45** | eliminated |
| 31. Explains clearly. | 3.08 (0.717)  **20** | 31 and 32 combined in second round  3.12 (0.730)  **16** |
| 32. Explains clearly during performance of a task which aspects are important and why.  Combination of items 31 and 32 in second round because of similarity of concept.  32. Explains clearly which aspects are important and why | 3.28 (0.614)  **14** |  |
| 33. Gives residents opportunities to think. | 2.92 (0.909)  **27** | eliminated |
| 34. Gives residents opportunities to practise. | 3.32 (0.627)  **11** | 3.36 (0.700)  **8** |
| 35. Has good teaching skills. | 2.68 (0.900)  **41** | eliminated |
| 36. Demonstrates commitment to improving teaching. | 2.68 (0.852)  **42** | eliminated |
| 37. Sets clear roles for residents. | 2.96 (0.935)  **25** | - 1. (0.680)   **20** |
| 38. Discusses goals with residents.  Reworded in response to panellists’ suggestions into:  Discusses training goals with residents during rotation. | 2.80 (0.913)  **36** | 2.62 (0.770)  **26** |
| 39. Clearly specifies what the resident is expected to know and do during the training period. | 2.80 (0.645)  **37** | eliminated |
| 40. Demonstrates skills for learner assessment/evaluation. | 2.60 (0.957)  **48** | eliminated |
| 41. Shares up-to-date knowledge of developments in the field. | 2.92 (0.909)  **28** | eliminated |
| 42. Encourages residents to discuss the care plan of each patient within the team.  Reworded into  Shows the importance of team work  and combined with item 50 because of similarity of concept. | 2.64 (0.995)  **46** | 3.28 (0.740)  **11** |
| 43. Is a scholar.  Reworded as:  Teaches residents how to conduct clinical research | 2.16 (0.987)  **52** | 2.84 (0.990)  **23** |
| 44. Is a good role model of doctor-patient relationships.  In second round item 44 was combined with item 46:  44. Is a good role model of doctor-patient relationships (treats patients with respect, etc.) | 3.04 (0.978)  **21** | 3.50 (0.660)  **3** |
| 45. Teaches effective communication skills. | 2.84 (0.898)  **33** | eliminated |
| 46. Treats patients with respect. | 3.64 (0.49)  **1** | combined with item 44 |
| 47. Is competent. | 2.94 (0.889)  **26** | eliminated |
| 48. Shows enthusiasm for medicine. | 2.88 (0.833)  **31** | eliminated |
| 49. Does not pretend to know all things. | 3.40 (0.707)  **8** | 3.20 (0.820)  **12** |
| 50. Makes an effort to establish good relations with medical staff.  Item reworded on panellist’s suggestion into:  Is a good role model of relationship with medical staff  and combined with item 42 because of similarity of concept. | 3.20 (0.764)  **17** | 3.36 (0.760)  **9** |
| 51. Maintains health, appearance and hygiene. | 2.60 (0.866)  **49** | eliminated |
| 52. I would like to work with this attending physician again. | 3.40 (0.816)  **9** | 3.20 (0.650)  **13** |
| **Items suggested by panellists in the first Delphi round** |  |  |
| 53. Shows social common sense. |  | 2.76 (0.830)  **24** |
| 54. Encourages consideration of psychosocial problems. |  | 3.04 (0.610)  **18** |
| Shows the importance of communication with staff | Not included due to similarity with item 50. | |
