# Supplementary material for: An instrument for evaluating clinical teaching in Japan: content validity and cultural sensitivity
Source: BMC Med Educ. 2014 Aug 28;14:179. doi: 10.1186/1472-6920-14-179 (PMC4167259; doi:10.1186/1472-6920-14-179)
Supplement: Supplementary file 4 — Additional file 4: The final validated instrument. (DOCX 22 KB) [file 12909_2014_1010_MOESM4_ESM.docx]

**Table 4**. The final validated instrument

| **Teaching evaluation sheet** | | | | | |
| --- | --- | --- | --- | --- | --- |
| **Hospital：**　　　　　 　　**Terms of rotation：**　 month **Course：** | | | | | |
| **Teacher’s Name：**　　　　　　　　　　　**Date：**　　　　　　　　　(dd/mm/yy) | | | | | |
| **Please evaluate your teacher .　Encircle　the　appropriate　response on　the　rating　scale.** | | | | | |
| **My clinical teacher:** | Strongly disagree | Disagree | Neutral | Agree | Strongly agree |
| 1. Shows enthusiasm for teaching. | 1 | 2 | 3 | 4 | 5 |
| 1. Is accessible. | 1 | 2 | 3 | 4 | 5 |
| 1. Encourages residents to call him or her at any time for any reason. | 1 | 2 | 3 | 4 | 5 |
| 1. Displays reasoning processes. | 1 | 2 | 3 | 4 | 5 |
| 1. Provides sufficient support. | 1 | 2 | 3 | 4 | 5 |
| 1. Stimulates residents' interest in learning and/or subject. | 1 | 2 | 3 | 4 | 5 |
| 1. Treats residents with respect.   (does not make personal criticisms, does not teach residents in an angry voice, does not provide negative feedback in front of others, etc.) | 1 | 2 | 3 | 4 | 5 |
| 1. Actively involves residents in patient care. | 1 | 2 | 3 | 4 | 5 |
| 1. Sets clear roles for residents. | 1 | 2 | 3 | 4 | 5 |
| 1. Stimulates residents to think critically when solving a problem. | 1 | 2 | 3 | 4 | 5 |
| 1. Is a good role model for relationships with medical staff. | 1 | 2 | 3 | 4 | 5 |
| 1. Is a good role model for doctor-patient relationships   (treats patients with respect , etc.) | 1 | 2 | 3 | 4 | 5 |
| 1. Is a good clinical supervisor at all times. | 1 | 2 | 3 | 4 | 5 |
| 1. Gives residents opportunities to practise. | 1 | 2 | 3 | 4 | 5 |
| 1. Does not pretend to know all things. | 1 | 2 | 3 | 4 | 5 |
| 1. Demonstrates the importance of safety. | 1 | 2 | 3 | 4 | 5 |
| 1. Thinks speculatively about areas of uncertainty with residents. | 1 | 2 | 3 | 4 | 5 |
| 1. Explains clearly which aspects are important and why. | 1 | 2 | 3 | 4 | 5 |
| 1. Gives concrete indications about what should be improved. | 1 | 2 | 3 | 4 | 5 |
| 1. Contributes additional clinical information or advice about diagnosis and management plan for each active problem. | 1 | 2 | 3 | 4 | 5 |
| 1. Encourages residents to consider psychosocial problems. | 1 | 2 | 3 | 4 | 5 |
| 1. Teaches residents how to conduct clinical research. | 1 | 2 | 3 | 4 | 5 |
| 1. Discusses clear training goals with residents during rotations. | 1 | 2 | 3 | 4 | 5 |
| 1. Shows social common sense. | 1 | 2 | 3 | 4 | 5 |
| 1. I would like to work with this teacher again. | 1 | 2 | 3 | 4 | 5 |
